# Irregular vascular pattern by contrast‐enhanced ultrasonography and high serum Lens culinaris agglutinin‐reactive fraction of alpha‐fetoprotein level predict poor outcome after successful radiofrequency ablation in patients with early‐stage hepatocellular carcinoma

**DOI:** 10.1002/cam4.932

**Published:** 2016-10-17

**Authors:** Hitomi Takada, Kaoru Tsuchiya, Yutaka Yasui, Natsuko Nakakuki, Nobuharu Tamaki, Shoko Suzuki, Hiroyuki Nakanishi, Jun Itakura, Yuka Takahashi, Masayuki Kurosaki, Yasuhiro Asahina, Nobuyuki Enomoto, Namiki Izumi

**Affiliations:** ^1^ Department of Gastroenterology and HepatologyMusashino Red Cross HospitalTokyoJapan; ^2^ First Department of Internal MedicineUniversity of YamanashiYamanashiJapan; ^3^ Division of Gastroenterology and HepatologyDepartment of Internal Medicine IIIMedical University of ViennaViennaAustria; ^4^ Liver Disease ControlTokyo Medical and Dental UniversityTokyoJapan

**Keywords:** Alpha‐fetoprotein, contrast‐enhanced ultrasonography, hepatocellular carcinoma, intrasubsegmental recurrence, poor survival, radiofrequency ablation

## Abstract

Radiofrequency ablation (RFA) is considered the most effective treatment for early‐stage hepatocellular carcinoma (HCC) patients unsuitable for resection. However, poor outcome after RFA has occasionally been reported worldwide. To predict such an outcome, we investigated imaging findings using contrast‐enhanced ultrasonography (CEUS) with Sonazoid and serum tumor markers before RFA. This study included 176 early‐stage HCC patients who had initially achieved successful RFA. Patients were examined using CEUS; their levels of alpha‐fetoprotein (AFP), Lens culinaris agglutinin‐reactive fraction of AFP (AFP‐L3), and des‐gamma‐carboxy prothrombin before RFA were measured. Sonazoid provided parenchyma‐specific contrast imaging and facilitated tumor vascular architecture imaging through maximum intensity projection (MIP). Kaplan–Meier analysis examined cumulative rates of local tumor progression, intrasubsegmental recurrence, and survival; factors associated with these were determined with Cox proportional hazards analysis. Local tumor progression (*n* = 15), intrasubsegmental recurrence (*n* = 46), and death (*n* = 18) were observed. Irregular pattern in MIP classification and serum AFP‐L3 level (>10%) before RFA were identified as independent risk factors for local tumor progression and intrasubsegmental recurrence. These two factors were independently associated with poor survival after RFA (irregular pattern in MIP: hazard ratio, (HR) = 8.26; 95% confidence interval, (CI) = 2.24–30.3; *P *=* *0.002 and AFP‐L3 > 10%: HR = 2.94; 95% CI = 1.09–7.94; *P *=* *0.033). Irregular MIP pattern by CEUS and high level of serum AFP‐L3 were independent risk factors for poor outcome after successful RFA. The Patients with these findings should be considered as special high‐risk group in early‐stage HCC.

## Introduction

Liver cancer, predominantly hepatocellular carcinoma (HCC), is the second and sixth most deadly cancer occurring in men and women, respectively, worldwide 1. Surgical resection can be effective for patients with early‐stage HCC. However, in real practice, only selected patients can undergo surgical resection because of restrictions associated with liver function, complications, and age. Radiofrequency ablation (RFA) is another effective and safe noninvasive treatment for early‐stage HCC. However, local tumor progression after RFA has been reported as an RFA limitation, with rates ranging from 2.4% to 23.3% in previous studies 2, 3, 4, 5. Local tumor progression, intrasubsegmental recurrence, and poor survival after successful RFA are associated with the biological malignant potential of the original tumor, and it is very important to evaluate the potential before RFA. Although pathological diagnosis can be helpful, it is difficult to perform tumor biopsy for all patients before RFA. Recent studies have reported contrast‐enhanced ultrasonography (CEUS) with Sonazoid could predict histological differentiation and portal vein invasion in patients with HCC 6, 7. CEUS is noninvasive and has few contraindications. Perflubutane microbubbles, Sonazoid (Daiichi‐Sankyo, Tokyo, Japan; GE Healthcare, Little Chalfont, UK) is a second‐generation ultrasound contrast agent that consists of a lipid‐stabilized suspension of perfluorobutane gas microbubbles within a hard shell of phosphatidyl‐serine. It provides both real‐time vascular‐phase imaging and parenchyma‐specific liver imaging called Kupffer imaging. Parenchyma‐specific liver imaging is enabled by the uptake of Sonazoid microbubbles by Kupffer cells in the reticuloendothelial system of the liver 8, 9. As predictive markers in patients with HCC, tumor markers including those for alpha‐fetoprotein (AFP), Lens culinaris agglutinin‐reactive fraction of AFP (AFP‐L3), and des‐gamma‐carboxy prothrombin (DCP) have been reported in many past studies 10, 11, 12, 13, 14. AFP‐L3 has been widely used for HCC diagnosis and follow‐up surveillance, and its prognostic value has been reported in a recent meta‐analysis 15. The measurement of serum tumor markers in patients with HCC is also noninvasive and more objective than imaging results. Hence, we investigated CEUS imaging and serum tumor markers before RFA to predict poor outcome after successful RFA in patients with early‐stage HCC.

## Materials and Methods

### Patients

**Figure 1:**
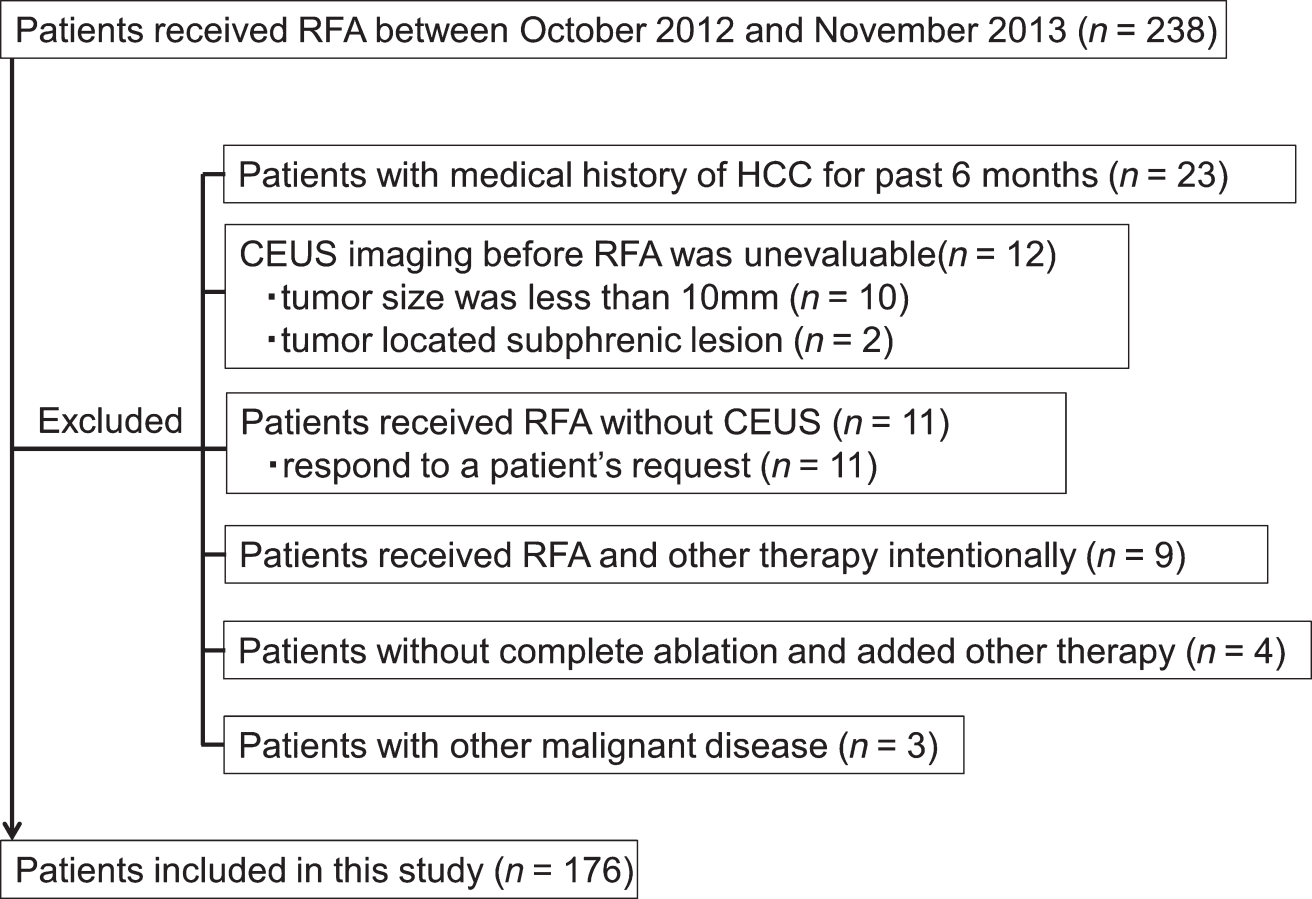
Flow diagram of case identification process. HCC; hepatocellular carcinoma, RFA;radiofrequency ablation, CEUS;contrast‐enhanced ultrasonography.

We performed CEUS with Sonazoid in 650 consecutive patients appearing at the Musashino Red Cross Hospital for screening or diagnosis of hepatic nodules between October 2012 and November 2013. Of these, 238 patients were diagnosed with HCC and underwent RFA. Indications for RFA were as follows: solitary HCC tumor ≤50 mm or three or fewer lesions, none >30 mm; without major vascular or biliary invasion; a total bilirubin concentration <2.5 mg/dL; platelet count >25.0 × 10^3^/mm^3^; and prothrombin activity >40%. As a result, 176 of 238 patients who had received RFA were eligible for this study based on the inclusion criteria. The inclusion criteria of this study were as follows: (1) patients with no medical history of HCC for the past 6 months before RFA; (2) the CEUS imaging before RFA was evaluable, and AFP‐L3 was measured before RFA; (3) RFA was judged to be successful by dynamic computed tomography (CT) or dynamic magnetic resonance imaging (MRI). Successful HCC ablation was defined as hypoattenuation of the entire tumor; and (4) follow‐up period was >6 months after RFA. The screening process is detailed in Figure 1. Written‐informed consent was obtained from all patients. This study was approved by the Ethics Committee of the Musashino Red Cross Hospital and was conducted in accordance with the Declaration of Helsinki.

### HCC diagnosis

HCC diagnosis was confirmed by typical radiographic findings on dynamic CT and dynamic MRI or by needle biopsy. For triple‐phase dynamic CT scans, arterial, portal, and equivalent phases were set at 35, 70, and 150 sec, respectively, after the injection of the contrast agent. Board‐certified radiologists diagnosed HCC on the basis of typical patterns, such as an early‐phase hyperattenuation area or a late‐phase hypoattenuation area on dynamic CT or MRI. Early‐phase hyperattenuation means the nodule was enhanced in arterial phase of dynamic CT or MRI. Nodule biopsy was performed when a definitive diagnosis was achieved by imaging techniques. The final diagnosis was confirmed by certified pathologists who were unaware of the patient's clinical data. We used the BCLC staging system 16 and in this system, early‐stage HCC was defined as single or multiple HCCs within 3 nodules ≤3 cm. All of the patients including this study were treated with RFA, therefore when a patient had a single HCC, we only included the patient whose tumor size was within 5 cm. In this study, early‐stage HCC means single within 5 cm or multiple HCCs within 3 nodules ≤3 cm. It is the same definition as the Milan criteria 17.

### CEUS

**Figure 2:**
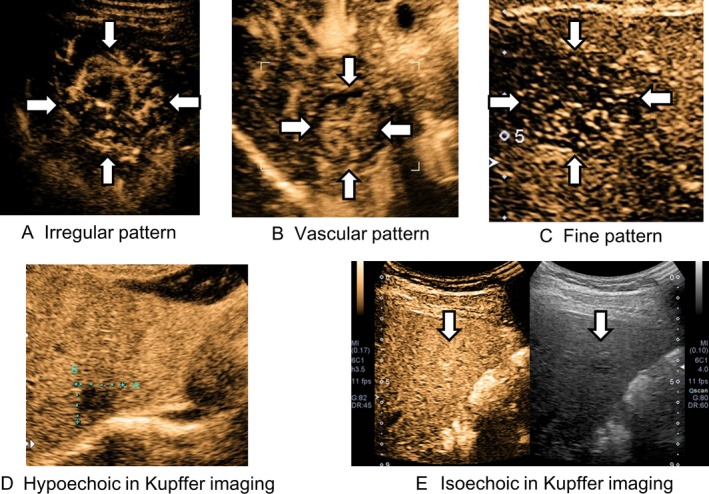
Images of MIP classification and Kupffer imaging. (A) Irregular pattern in MIP classification. (B) Vascular pattern in MIP classification. (C) Fine pattern in MIP classification. (D) Hypoechoic pattern in the Kupffer imaging in CEUS with Sonazoid. (E) Isoechoic pattern in the Kupffer imaging in CEUS with Sonazoid. MIP, maximum intensity projection; CEUS, contrast‐enhanced ultrasonography.

CEUS scans were obtained by two hepatologists, including a sonographic specialist with at least 5 years of experience in CEUS. The intravenous sonographic contrast agent, Sonazoid, was used in all patient investigations. We injected a 0.5‐mL suspension at a speed of 1 mL/sec, followed by immediate flushing with 5–10 mL of normal saline via a peripheral venous line. US equipment was Aplio500 (Toshiba, Medical Systems, Tokyo, Japan) with convex (PVT‐375 BT) or microconvex probe (PVT‐382 BT). The region of interest was observed continuously for approximately 90 sec from the time of injection. The arterial phase was timed for 45 sec after the completion of the flash. After portal phase, we examined the MIP pattern using microflow imaging. MIP can help visualize this fine vascular structure and was first introduced by Sugimoto et al. 11. The MIP pattern was classified into one of the following three patterns defined according to recent reports 6, 18 (Fig. 2): (1) fine pattern, wherein tumor vessels were not clearly visualized, and only fine vessels were visualized; (2) vascular pattern, wherein tumor vessels were visualized clearly; and (3) irregular pattern, wherein tumor vessels were thick and irregular. Approximately 10 min after the injection via the peripheral venous line, the liver was scanned again to observe Kupffer imaging. Patterns observed in Kupffer imaging were classified as follows: (1) hyper or isoechoic pattern, (2) hypoechoic pattern.

### Measurement of tumor markers

Serum AFP, AFP‐L3, and DCP were measured before RFA. Serum AFP levels were determined by chemiluminescent immunoassays (Siemens Immulyze AFP 20 IV; Mitsubishi Chemical Medience, Tokyo, Japan and Architect AFP EX; Abbott Japan, Tokyo, Japan). Serum AFP‐L3 levels were expressed as a percentage of the total AFP (AFP‐L3/total AFP × 100) using a liquid‐phase binding assay (LBA; Wako AFP‐L3 or *μ*TAS Wako AFP‐L3, Wako Pure Chemical Industries, Osaka, Japan). Serum DCP levels were determined by chemiluminescent enzyme immunoassay (Lumipulse PIVKA‐II; Eisai, Tokyo, Japan). In total, three patients were treated with warfarin, and we excluded these patients from DCP‐related analyses.

### RFA procedure

RFA was performed under local anesthesia with the percutaneous approach. We used an internally water‐cooled 17‐gauge cooled‐tip electrode with an impedance‐controlled generator (Cosman generator, Cool‐Tip system, Radionics, Burlington, MA). Ultrasonography was performed with Aplio 500 systems (Toshiba Medical Systems, Tokyo, Japan). When the target nodule was >20 mm in diameter, we performed multiple needle insertions and multiple ablations of 1 nodule.

### Assessment of treatment efficacy and follow‐up

Dynamic CT or dynamic MRI scan with a section thickness of 2.5–5 mm was performed to evaluate the efficacy of ablation 1–3 days after RFA. Complete HCC ablation (successful RFA) was defined as hypoattenuation of the entire tumor. Blood was sampled every 1–3 months and tested for indicators of liver function and tumor markers, including AFP, AFP‐L3, and DCP. A dynamic CT scan or dynamic MRI was scheduled every 3–4 months. Local tumor progression was defined as a new appearance of the enhancement within or at the periphery of the original tumor. Intrahepatic recurrence was divided into intrasubsegmental and extrasegmental recurrence. Intrasubsegmental recurrence was defined as any new tumor that occurred in the liver separate from the ablated area but within the same liver segment as RFA site, according to Couinaud's liver segment. When a tumor was located on some subsegments, the subsegment where the major part of the tumor was present was adopted. Time to recurrence was estimated from the date of the first RFA to the date the presence of the tumor was diagnosed by imaging. The end of follow‐up was death or latest medical attendance up to August 9, 2015.

### Statistical analysis

Primary endpoints were local tumor progression and intrasubsegmental recurrence and secondary endpoint was death after successful RFA. The cumulative rates of local tumor progression, intrasubsegmental recurrence, and survival after successful RFA were determined with the Kaplan–Meier method. The risk factors associated with local tumor progression, intrasubsegmenal recurrence, and poor survival after successful RFA were identified with Cox proportional hazards regression model. Multivariate analysis was performed using the Cox proportional hazards model to identify independent prognostic factors for local tumor progression, intrasubsegmental recurrence, and poor survival after successful RFA. Covariate hazards of survival and recurrence were age at the RFA, HCV RNA status, HBs (Hepatitis‐B Surface) antigen and HBc (Hepatitis‐B Core) antibody status, platelet counts, serum albumin and total bilirubin level, prothrombin time, pretreatment AFP level, pretreatment AFP‐L3 index, pretreatment DCP level, and irregular vascular pattern by CEUS. All statistical analyses were performed using statistical software (SPSS 11.0 for Windows, SPSS Inc., Chicago, IL). A *P *< 0.05 was considered statistically significant.

## Results

Patient characteristics are shown in Table 1. 139 patients showed positive for anti‐HCV antibody, 73 patients of them received anti‐HCV therapy before RFA, and 19 patients achieved sustained viral response. All anti‐HCV therapies contained regular or pegylated interferon. Twelve patients showed positive for HBs antigen and 68 patients showed positive for anti‐HBc antibody. The data of prothrombin time and DCP level for three patients treated with warfarin have been excluded from analysis. In total, 162 were hypervascular, and 14 nodules were detected with hypovascular HCC. Of the latter, eight hypovascular nodules were diagnosed by percutaneous needle biopsy; one was detected with early‐stage HCC, three nodules were well‐differentiated, one was moderately differentiated, and one had poorly differentiated carcinoma. The other six nodules showed hypodensity in the hepatobiliary phase of gadolinium–ethoxybenzyl–diethylenetriamine penta‐acetic acid (Gd–EOB–DTPA)‐enhanced MRI and an increase in tumor size in the resent 3 months. Irregular pattern in MIP classification in CEUS was diagnosed in six nodules, and 44 nodules showed a higher serum AFP‐L3 index (>10%) before RFA.

**Table 1:** Characteristics the 176 patients with early‐stage HCC

Characteristics	Value
Age, years	74 (44–90)
Duration of follow‐up, months	26 (7.4–35)
Sex, [*n* (%)]	
Male	103 (59)
Female	73 (41)
Clinical and laboratory data	
AFP, median (range), ng/mL	11.1 (2–11000)
AFP‐L3 index, median (range), %	4.5 (0.5–83.6)
DCP, median (range), mAU/mL	21 (10–3790)
Child–Pugh class [*n* (%)]	
A	167 (95)
B	9 (5)
Serum albumin, median (range), g/dL	3.7 (2.3–5.1)
Serum total bilirubin, median (range), mg/dL	0.8 (0.2–8)
Prothrombin time, median (range), %	91.4 (47.2–120)
Pathology	
Maximum HCC diameter, mm, [*n* (%)]	
<20 mm	128 (73)
≥20 mm	48 (27)
Number of HCC nodules, [*n* (%)]	
Single	125 (71)
Multiple	51 (29)
Lymph node involvement (%)	0
Metastasis (%)	0
Treatment curse	
Naïve	44 (25)
Recurrent	132 (75)
Major associated liver diseases, [*n* (%)]	
HBV	12 (6.8)
HCV	139 (79)
Alcohol	11 (6.3)
NASH	16 (9.1)
unknown (%)	3 (1.7)

DCP, des‐gamma‐carboxy prothrombin.

**Figure 3:**
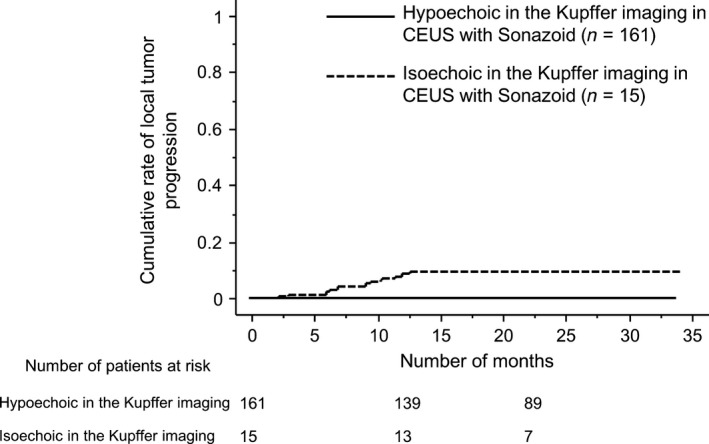
Kaplan–Meier plots for local tumor progression after successful RFA, according to an echoic pattern in the Kupffer phase in CEUS before treatment.

The median duration of follow‐up was for 25.6 months (7.4–34.8 months). Local tumor progression was observed in 15 nodules (8.5%), and all cases presented hypervascular nodules with low echoic pattern in Kupffer imaging before RFA. No local recurrence was observed in nodules with isoechoic pattern in Kupffer imaging (*n* = 15) before RFA. (Fig. 3). Intrasubsegmental recurrence occurred in 46 nodules (26.1%), and extrasegmental recurrence was observed in 39 nodules (22.2%). Distal metastasis was observed in 23 patients and all the patients received RFA as a treatment for recurrence of HCC. In total, 18 patients died within the observation period. The cumulative survival rates after RFA at Year 1 and 2 were 97.1% and 90.6%, respectively.

The sensitivity, specificity, positive predictive value (PPV) and negative predictive value (NPV) of irregular vascular pattern by CEUS for local recurrence is 13.3%, 96.9%, 28.6%, and 92.3%, and those of high AFP‐L3 level is 60.0%, 78.3%, 20.5%, and 95.5%. The sensitivity, specificity, PPV, and NPV of irregular vascular pattern by CEUS for intrasubsegmental recurrence is 8.7%, 97.7%, 57.1%, and 75.1%, and those of high AFP‐L3 level is 37.0%, 79.2%, 38.6%, and 78.0%.

### Risk factors for local tumor progression after successful RFA

Univariate analysis showed that irregular pattern in MIP classification and a higher AFP‐L3 level (>10%) before RFA were significantly associated with local tumor progression. Based on multivariate analysis, irregular pattern in MIP classification (HR  = 5.46; 95% confidence interval, CI = 1.23–24.4; *P *=* *0.025) and serum AFP‐L3 index of >10% (HR = 5.10, 95% CI = 1.82–14.3, *P *=* *0.002) before RFA were identified as independent risk factors for local tumor progression (Table 2).

**Table 2:** Cox proportional hazards analysis for risk factors associated with local tumor progression after RFA (*n* = 176)

Factors	Univariate analysis	Multivariate analysis
	*P* value (HR, 95% CI)	*P* value (HR, 95% CI)
Age	0.38	
HCV antibody positive	0.28	
HCV RNA status (SVR)	0.59	
HBs antigen positive	0.44	
HBc antibody positive	0.47	
Platelet counts (×10^3^/*μ*L)	0.67	
Serum albumin level (g/dL)	0.46	
Serum total bilirubin level (g/dL)	0.65	
Prothrombin time (%)	0.77	
AFP level (ng/mL)	0.49	
AFP‐L3 index >10%	0.002 (5.10, 1.81–14.3)	0.002 (5.10, 1.82–14.3)
DCP level (mAU/mL)	0.54	
Tumor size (cm)	0.19	
MFI irregular pattern	0.027 (5.38, 1.21–23.8)	0.025 (5.46, 1.23–24.4)

HR, hazard ratio; CI, confidence interval; SVR, sustained viral response; DCP, des‐gamma‐carboxy prothrombin.

### Risk factors for intrasubsegmental recurrence after successful RFA

Univariate analysis showed that tumor size, irregular pattern in MIP classification, and a higher AFP‐L3 level (>10%) before RFA were significantly associated with intrasubsegmental recurrence. Based on multivariate analysis, irregular pattern in MIP classification (HR = 3.05, 95% CI = 1.02–9.17, *P *=* *0.047); and serum AFP‐L3 index of >10% (HR = 2.44, 95% CI = 1.33–4.46, *P *=* *0.004) were identified as independent risk factors for subsegmental recurrence after successful RFA (Table 3).

**Table 3:** Cox proportional hazards analysis for risk factors associated with intrasubsegmental recurrence after RFA (*n* = 176)

Factors	Univariate analysis	Multivariate analysis
	*P* value (HR, 95% CI)	*P* value (HR, 95% CI)
Age	0.52	
HCV antibody positive	0.10	
HCV RNA status (SVR)	0.08	
HBs antigen positive	0.26	
HBc antibody positive	0.21	
Platelet counts (×10^3^/*μ*L)	0.47	
Serum albumin level (g/dL)	0.21	
Serum total bilirubin level (g/dL)	0.75	
Prothrombin time (%)	0.91	
AFP level (ng/mL)	0.79	
AFP‐L3 index >10%	0.003 (2.49, 1.36–4.55)	0.004 (2.44, 1.33–4.46)
DCP level (mAU/mL)	0.48	
Tumor size (cm)	0.02 (1.04, 1.01–1.09)	0.074
MFI irregular pattern	0.04 (4.63, 1.64–13.0)	0.047 (3.05, 1.02–9.17)

HR, hazard ratio; CI, confidence interval; SVR, sustained viral response; AFP, alpha‐fetoprotein; DCP, des‐gamma‐carboxy prothrombin; RFA, Radiofrequency ablation.

### Risk factors for multicentric recurrence after successful RFA

As an evaluation of risk factors associated with multicentric recurrences after successful RFA, we focused on the patients who had shown the extrasegmental recurrences. 36 patients showed only extrasegmental recurrences and in 32 patients both intra‐ and extrasegmental recurrence were observed at the same time. In univariate and multivariate analyses, pretreatment AFP‐L3 level and irregular vascular pattern by CEUS were significant independent risk factors associated with multicentric recurrence for HCC (irregular pattern in MIP: HR = 3.82; 95% CI = 1.52–9.53; *P *=* *0.004 and AFP‐L3 > 10%: HR = 1.72; 95% CI = 1.02–2.93; *P *=* *0.043).

### Risk factors for poor survival after successful RFA

Univariate analysis showed that serum albumin, serum total bilirubin, irregular pattern in MIP classification, and a higher AFP‐L3 level (>10%) before RFA were significantly associated with poor survival. Based on multivariate analysis, pretreatment total bilirubin (HR = 1.69, 95% CI = 1.12–2.56, *P *=* *0.0013); irregular pattern in MIP classification (HR = 8.26, 95% CI = 2.24–30.3, *P *=* *0.002); pretreatment serum AFP (HR = 1.00, 95% CI = 1.000–1.001); and pretreatment serum AFP‐L3 index of >10% (HR = 2.94, 95% CI = 1.09–7.93, *P *=* *0.033) were identified as independent risk factors for poor survival (Table 4). Furthermore, we analyzed the prognostic factors associated with survival using the posttherapeutic factors including local recurrence, intrasubsegmental recurrence, and distal metastasis. As a result, intrasubsegmental recurrence was an independent prognostic factor associated with survival (HR = 2.82, 95% CI = 1.11–7.14, *P *=* *0.029), and the other two factors, local recurrence and distal metastasis, were not significantly associated with overall survival in this study.

**Table 4:** Cox proportional hazards analysis for risk factors associated with overall survival (*n* = 176)

Factors	Univariate analysis	Multivariate analysis
	*P* value (HR, 95% CI)	*P* value (HR, 95% CI)
Age	0.17	
HCV antibody positive	0.30	
HCV RNA status (SVR)	0.95	
HBs antigen positive	0.64	
HBc antibody positive	0.08	
Platelet counts (×10^3^/*μ*L)	0.048 (1.13,1.00–1.28)	0.23
Serum albumin level (g/dL)	0.045 (2.56,1.02–6.41)	0.53
Serum total bilirubin level (g/dL)	0.002 (1.76,1.23–2.51)	0.013 (1.69,1.12–2.56)
Prothrombin time (%)	0.13	
AFP level (ng/mL)	<.0001 (1.00,1.000–1.001)	0.002 (1.00,1.000–1.001)
AFP‐L3 index >10%	0.01 (3.33,1.32–8.40)	0.033 (2.94, 1.09–7.93)
DCP level (mAU/mL)	<.0001(1.00, 1.001–1.002)	0.60
Tumor size (cm)	0.42	
MFI irregular pattern	0.001 (7.75, 2.23–27.0)	0.002 (8.26,2.24–30.3)

HR, hazard ratio; CI, confidence interval; SVR, sustained viral response; AFP, alpha‐fetoprotein; DCP, des‐gamma‐carboxy prothrombin.

### The changes in tumor markers after successful RFA

We investigated the changes in tumor markers after RFA. Despite of achieving successful RFA, 48 of 176 patients (27.2%) showed increased serum AFP level after RFA. About posttreatment AFP‐L3 index, 41 of 176 (23.3%) patients did not show the decrease and in 54 of 176 (30.7%) patients, serum DCP were increased after successful RFA. In the patients whose all three tumor markers (AFP, AFP‐L3, and DCP) had increased after successful RFA (*n* = 9), intra‐ and extrasubsegmental recurrence for HCC were significantly higher than the other patients (subsegmental recurrence: HR = 3.91; 95% CI = 1.68–9.10; *P *=* *0.002 and extrasubsegmental recurrence: HR = 2.33; 95% CI = 1.04–5.21; *P *=* *0.040). There was no significant difference in local tumor progression.

## Discussion

RFA is considered to be the first‐line treatment option for patients with early‐stages HCC unsuitable for surgical resection. However, it was reported that even in patients with very early‐ or early‐stage HCC, 79.3% of all patients had moderate or poorly differentiated HCC and 17.0% of all patients had microvascular invasion, as indicated by a recent study including 324 patients treated with resection 19. Poor outcome including local tumor progression, intrasubsegmental recurrence, and short survival after successful RFA initially have biological malignant potential including pathological differentiation (poorly differentiated HCC) and microvascular invasion. In particular, microvascular invasion, which is usually impossible to diagnose without resection, was an independent prognostic factor for survival after resection in very early‐ or early‐stage HCC 19. Therefore, it is very important for the patients with early‐stage HCC to evaluate the biological malignant potential before treatment. In this study, we performed CEUS with Sonazoid and measured well‐known tumor markers, including serum AFP, AFP‐L3, and DCP, before RFA. Tanaka et al. 6. reported that imaging diagnosis using CEUS with Sonazoid facilitated the estimation of histological differentiation and portal vein invasion in HCC. They used the grading system based on the combined assessment of Kupffer imaging and MIP pattern; we have used the same MIP classification in this study. In addition to pathological findings, Sato et al. 7. reported that vascular patterns in contrast‐enhanced intraoperative ultrasonography (CEIOUS) were distinctly identifiable by gene expression profiling associated with cellular proliferation of HCC. They performed CEIOUS with Sonazoid, and the CEIOUS images of HCC vascular patterns were classified as reticular HCC or thunderbolt HCC. The thunderbolt pattern showed findings similar to the irregular pattern in the recent classification 6 and thunderbolt HCC was significantly correlated with a higher AFP level, tumor size, histological differentiation, and portal vein invasion; these patients demonstrated a significantly poorer prognosis for both recurrence‐free survival and overall survival.

AFP‐L3 is one of the tumor markers for HCC and in many studies, it was reported that recurrence‐free survival in patients with a high AFP‐L3 level before RFA or resection was lower than that in patients with a low AFP‐L3 level. Cheng et al. 15. presented in their meta‐analysis that high pretreatment serum AFP‐L3 levels indicated a poor prognosis, and AFP‐L3 may have significant prognostic value in patients with HCC having a low AFP concentration.

Local tumor progression after RFA was investigated in many studies, and the rate of progression differed in all studies 2, 3, 4, 5, 20. Siina et al. 3. showed that 5‐ and 10‐year local tumor progression rates were both 3.2%, and the serum DCP level alone was significantly related to local tumor progression. Kono et al. 20. reported the outcome of RFA in patients with 2‐cm or smaller HCC. In their report, the cumulative local tumor recurrence rate in all patients at 1, 2, and 3 years was 9%, 19%, and 19%, respectively. Based on multivariate analysis, one of the independent risk factors for local tumor progression after RFA was irregular gross type diagnosed by imaging. Their study demonstrated that local tumor progression after RFA was associated with ablative margin, and an advanced evaluation of original tumor by imaging was necessary even in patients with 2‐cm or smaller HCC. Sasaki et al. 21. investigated the influence of the grade of histological differentiation on the outcome of liver resection for HCC 2 cm or smaller in size. The 3‐ and 5‐year recurrence‐free survival rates for poorly differentiated HCCs were 39% and 29%, respectively; these were significantly worse than those for the not poorly differentiated HCCs (64% and 50%, respectively; *P *<* *0.01). In poorly differentiated HCCs, anatomical resection resulted in a significantly lower recurrence rate than nonanatomical resection, and a wider resection margin did not significantly reduce the recurrence rate. In our study, irregular MIP pattern and a high AFP‐L3 level were significantly associated with local tumor progression, intrasubsegmental recurrence, and poor survival after successful RFA. HCCs with such findings should be treated with anatomically curative treatment rather than with a wide safety ablative margin.

In this study, we evaluated the malignant potential for HCC by both imaging modality and serum tumor markers. Recently, Wang et al. 22. reported about predictive value of conventional and contrast‐enhanced ultrasonography in early recurrence of HCC. They concluded that a preoperative serum AFP level ≥400 ng/mL, tumor diameter ≥5 cm and “fast wash‐out” enhancement pattern by CEUS are independent risk factors for early recurrence of HCC after surgical resection. As other reports using both imaging modality and serum tumor marker, Min et al. 23. concluded that log AFP, tumor size, and intratumoral fat were independent factors associated with microvascular invasion in patients with HCC. In this study intratumoral fat was evaluated by gadoxetic acid‐enhanced MR imaging. In these two studies, AFL‐L3 was not determined und in our study, tumor size was not a significant factor associated with poor prognosis because we only included the patients whose tumor size were not bigger than 5 cm.

Kobayashi et al. 24. reported that Fluorine 18 Fluorodeoxyglucose Positron Emission Tomography/Computed Tomography and AFP‐L3 might be useful for predicting microvascular invasion in small HCC, and the combination of the 2 factors provided reliable assessment for selection of suitable hepatic resection and liver transplantation candidates. Thus the evaluation of malignant potential in patients with HCC using both tumor markers and imaging modalities has been becoming an effective analysis.

In clinical setting, as pretreatment factors, serum T‐Bil level, serum AFP level, serum AFP‐L3 level >10%, and CEUS pattern were significant independent factors associated with poor survival. The results means that both preserved liver function and lower biological malignant potential of the tumor are very important for long survival. We only included the patients within the Milan criteria, so that the tumor size was not a significant factor for survival in this study.

Tateishi et al. 25. reported that the risk factors related to intrasubsegmental recurrence after RFA were tumor size, AFP levels, platelet count, and anti‐HCV antibody positivity. The results of our study were not similar to those reported in the past report because in the past study, there was no imaging and pathological data collection before RFA. In addition, their cut‐off value for AFP‐L3 level was 15%. However, in the systematic review and meta‐analysis of AFP‐L3, the most frequently used cut‐off value of AFP‐L3 was 10% 15.

Another novel finding of this study was that there was no local tumor progression in patients with isoechoic Kupffer imaging. In previous studies, most HCCs revealed hypoechoic pattern in the Kupffer imaging. Tanaka et al. 6. reported that only 5 of 72 HCCs in their study showed isoechoic pattern in Kupffer imaging, 4 of them were well‐differentiated HCC, and 1 of them was moderately differentiated HCC. None of them was poorly differentiated HCC or showed an irregular pattern of MIP classification. The sample size of the HCCs with isoechoic Kupffer imaging is small, but in such HCCs, local tumor progression after RFA is very rare and the recurrence rate may be the same between patients undergoing resection and those receiving RFA. This should be evaluated in a prospective randomized multicenter study.

The limitations of our study were that there was no long observation period and it was a single center study. However, the study provides the first report about poor outcome after successful RFA, focusing on the biological malignant potential of original tumor, using CEUS and tumor markers. The use of Sonazoid is not available in many countries; however, the arterial phase of CEUS can be evaluated with other contrast agents. These examinations are extremely noninvasive and uncomplicated; therefore, our findings have worldwide applicability.

Recently, local therapies for early‐stage HCC have become available, and there are some treatment techniques and modalities. Wang et al. 26. reported data about percutaneous cryoablation and RFA in patients with HCC in their large multicenter randomized controlled study. They reported that cryoablation resulted in a significantly lower local tumor progression than RFA. Xie et al. 27. demonstrated that the efficacy of RFA combined with transcatheter arterial chemoembolization (TACE) in 487 patients. In their analyses, tumor size and tumor marker were not significant risk factors for overall survival, and the 5‐year survival rate was 78.7%. In a meta‐analysis, it was reported that the combination of RFA and TACE was associated with a significantly higher overall survival rates and recurrence‐free survival rate 28. We reported risk factors for poor outcome after successful RFA in this study. A new therapeutic approach, other than RFA monotherapy, should be considered for the patients with these risk factors.

In conclusion, irregular MIP pattern in CEUS and high level of pretreatment serum AFP‐L3 were independent risk factors for local tumor progression, intrasubsegmental recurrence, and poor survival after successful RFA in patients with early‐stage HCC.

## Conflict of Interest

None declared.
